# Dysregulation of Gene Expression in the Artificial Human Trisomy Cells of Chromosome 8 Associated with Transformed Cell Phenotypes

**DOI:** 10.1371/journal.pone.0025319

**Published:** 2011-09-29

**Authors:** Hisakatsu Nawata, Genro Kashino, Keizo Tano, Kazuhiro Daino, Yoshiya Shimada, Hiroyuki Kugoh, Mitsuo Oshimura, Masami Watanabe

**Affiliations:** 1 Laboratory of Radiation Biology, Research Reactor Institute, Kyoto University, Osaka, Japan; 2 Advanced Molecular Imaging Center, Medical School, Oita University, Oita, Japan; 3 Experimental Radiobiology for Children's Health Research Group, Research Center for Radiation Protection, National Institute of Radiological Sciences, Chiba, Japan; 4 Department of Biomedical Science, Institute of Regenerative Medicine and Biofunction, Graduate School of Medical Sciences, Tottori University, Tottori, Japan; Florida State University, United States of America

## Abstract

A change in chromosome number, known as aneuploidy, is a common characteristic of cancer. Aneuploidy disrupts gene expression in human cancer cells and immortalized human epithelial cells, but not in normal human cells. However, the relationship between aneuploidy and cancer remains unclear. To study the effects of aneuploidy in normal human cells, we generated artificial cells of human primary fibroblast having three chromosome 8 (trisomy 8 cells) by using microcell-mediated chromosome transfer technique. In addition to decreased proliferation, the trisomy 8 cells lost contact inhibition and reproliferated after exhibiting senescence-like characteristics that are typical of transformed cells. Furthermore, the trisomy 8 cells exhibited chromosome instability, and the overall gene expression profile based on microarray analyses was significantly different from that of diploid human primary fibroblasts. Our data suggest that aneuploidy, even a single chromosome gain, can be introduced into normal human cells and causes, in some cases, a partial cancer phenotype due to a disruption in overall gene expression.

## Introduction

During cell division, errors in chromosomal segregation result in the loss or gain of chromosomes in daughter cells, which is referred to as aneuploidy. An extra or missing chromosome, known as trisomy and monosomy, respectively, is observed in people with developmental disabilities, mental retardation, and cancer. In addition, various types of cancer have karyotypes with complex numerical aberrations [1]–[3]. Although increasing evidence that aneuploidy is a hallmark of cancer, the causal relationship between aneuploidy and tumorigenesis remains unclear.

The addition of a single chromosome has been reported to have various transcriptional effects in several cell types [4]–[6]. An increase in the average transcriptional activity of a trisomic chromosome has been observed in trisomic primary mouse cells, human trisomic colorectal cancer cells, immortalized trisomic mammary epithelial cells, and acute myeloid leukemia (AML) cells (with an additional chromosome 8; derived from an AML patient). One chromosome affected not only the gene expression levels on the trisomic chromosome but also a large number of genes on other diploid chromosomes [5]. Apoptosis-regulating genes were significantly down regulated in AML cells containing an additional chromosome 8 that were derived from an AML patient [6].

Despite increased information about the characteristics of aneuploid human cells, the data reported thus far have been obtained from immortalized or cancer-derived cells. Normal human cells have a limited life span and ultimately enter a non-dividing state called senescence [7], [8]. It remains unclear whether aneuploidy renders cells immortal or if immortalization induces aneuploidy in cells. To determine the role(s) that aneuploidy plays in human cancer, it will be indispensable to design artificial aneuploid model cells derived from normal human cells.

Trisomy is a simple model of aneuploidy with the gain of a single chromosome. Trisomy of chromosome 8 is the most commonly observed trisomic chromosomal aberration, as has been demonstrated in fibroblastic/myofibroblastic tumors (Fig. S1) [9]–[12]. In this study, we used normal human diploid embryonic cells (HE35) as a donor for chromosome transfer, and chromosome 8 was chosen as the introduction chromosome. We succeeded in isolation of multiple clones that chromosome 8 became trisomic (trisomy 8 cells). All of the trisomy 8 cells expressed transformed cell-like phenotypes, such as a loss of contact inhibition, regrowth after senescence, and chromosome instability. The overall gene expression profile, determined by microarray analysis, was significantly different from that of the diploid HE35 cells. Our results suggest that aneuploidy is a key factor in tumorigenesis, as demonstrated by disrupted gene expression.

## Results

### Generation of human primary cells bearing three copies of chromosome 8 (trisomy 8 cells)

To generate the trisomy 8 cells, we used microcell-mediated chromosome transfer (MMCT) to introduce an additional chromosome 8 into HE35 cells of culture passage 7, which is a line of normal human diploid primary cells [13]. In this study, inactivated viral envelope proteins of the haemagglutinating virus of Japan (HVJ-E) were used. We isolated three independent clones (HE35tri8-1, -2, and -3) in 35-mm diameter dish, and cultured them by stepwise scale up until reaching confluence in three diameter 100 mm dishes (P100). Then cells were stocked at a total population doubling level of 30 (TPDL 30) for subsequent analysis.

**Figure 1:**
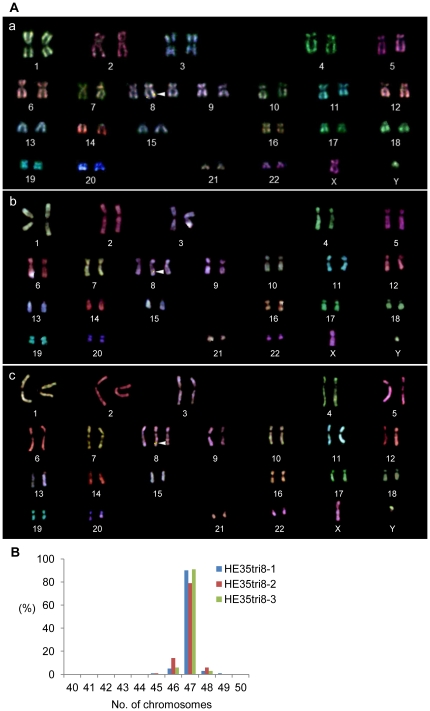
Generation of trisomic human cells of chromosome 8 (trisomy 8 cells). (A) Example of a Multicolor FISH analysis of a metaphase spread prepared from HE35tri8-1 at TPDL 30. The arrow indicates the transferred chromosome 8 (the yellow arrow is the neomycin-resistant gene in the transferred chromosome). (B) Chromosome distribution in the trisomy 8 cells.

To confirm that the extra chromosome was maintained in each cell clone, we performed Multicolor FISH, which identifies each chromosome by a unique fluorescent color (Fig. 1). The proportion of cells with three copies of chromosome 8 was 90% in HE35tri8-1 and HE35tri8-3 cells and 79% in HE35tri8-2 cells, indicating that each clone had an additional chromosome 8 (Fig. 1).

### Proliferation defects, loss of contact inhibition, and regrowth in the trisomy 8 cells

**Figure 2:**
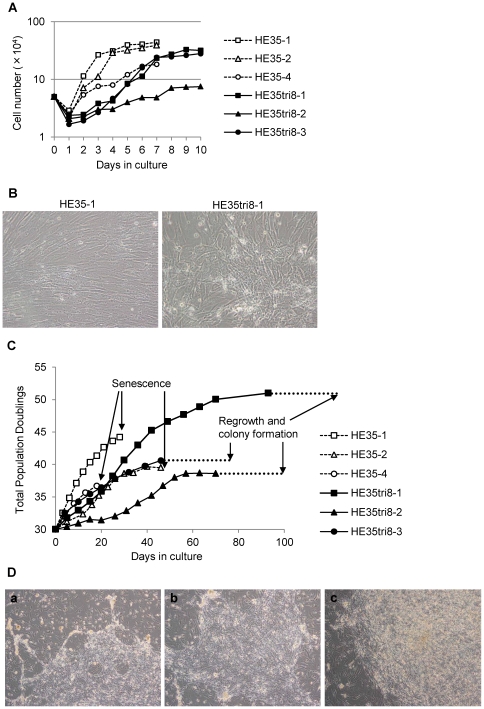
Proliferation defects, loss of contact inhibition, and regrowth in the trisomy 8 cells. (A) Proliferation defects in the artificial trisomy 8 cells. The diploid HE35 cells (open squares, open triangles, and open circles) and the trisomy 8 cells (solid squares, solid triangles, and solid circles) were plated and counted daily to detect changes in the cell numbers. (B) Loss of contact inhibition in the trisomy 8 cells (HE35tri8-1 at TPDL 30, HE35-1 at TPDL 30). (C) Cell growth of the cultured diploid HE35 cells and the trisomy 8 cells. (D) A small portion of the trisomy 8 cells regrew and formed colonies after 4–6 weeks of exhibiting a senescence-like state. (D-a) HE35tri8-1 colony, (D-b) HE35tri8-2 colony, (D-c) HE35tri8-3 colony.

Aneuploidy causes a proliferative disadvantage in mouse cells [4]. To investigate whether this phenotype could be observed in normal human cells, we examined the proliferative capacity of the artificial trisomy 8 cells in culture. Three artificial trisomy 8 cells had decreased proliferation compared to the diploid HE35 cells (Figs. 2A, S2A–B), indicating that the presence of an additional chromosome inhibits cell proliferation in culture.

Normal cell growth is arrested when cells contact each other in culture and in tissues. This phenomenon, known as contact inhibition, prevents uncontrolled cellular proliferation [14]. In contrast, transformed cells pile densely upon one another [15]. Growth arrest was observed upon cell contact for the normal diploid HE35 cells (Fig. 2B). However, the growth of the trisomy 8 cells (HE35tri8-1, -2, and -3) was not arrested when the cells made contact *in vitro*, and the cells piled densely on one another (Fig. 2B).

Normal human cells undergo a finite number of cell divisions and ultimately enter a non-dividing state called senescence, whereas neither transformed nor cancer cells undergo senescence when they become immortalized. To understand the effects of a single chromosome gain on senescence, we investigated whether the artificial trisomy 8 cells reached senescence and became immortalized. Three clones of normal diploid HE35 (HE35-1, -2, and -4) ultimately entered senescence (Figs. 2C, S3). On the other hand, the trisomy 8 cells (HE35tri8-1, -2, and -3) temporarily exhibited senescence-like characteristics, but after 4–6 weeks of this senescence-like phenotype, a small portion of the trisomy 8 cells had regrown and formed colonies (Figs. 2C and 2D, Table 1). These colonies were made with relatively small sized cells, and the characteristic of the colony morphology, such as piled-up and criss-cross, was the same as those of malignant cells (Fig. 2D). The colony grew up to approximately 5 mm in diameter, but was not able to finally get infinite growth ability.

**Table 1:** Frequency of colony formation by regrowth cells after senescence.

Cells	TPDL^a^	Number of regrowth colony per 1.5×10^6^ inoculated cells^b^
HE35-1	44.2	0
HE35-2	39.5	0
HE35-4	36.7	0
HE35tri8-1	51.0	5.0±2.3
HE35tri8-2	38.6	3.5±1.9
HE35tri8-3	40.6	1.0±1.1

a Total population doubling levels (TPDL) show the number of cell division that cells divided until reaching to senescence.

b Frequency of colony formation by regrowth cells in three P100 dishes after senescence. Briefly, 1.5×10^6^ cells were inoculated into three P100 dishes. Then, cells were incubated in a 100% humidified CO_2_ incubator at 37°C for 6 weeks with medium change every 3 days. Regrowth colonies were counted. Data show the mean ± S.D. of six independent experiments.

### DNA damage and chromosomal aberrations

**Figure 3:**
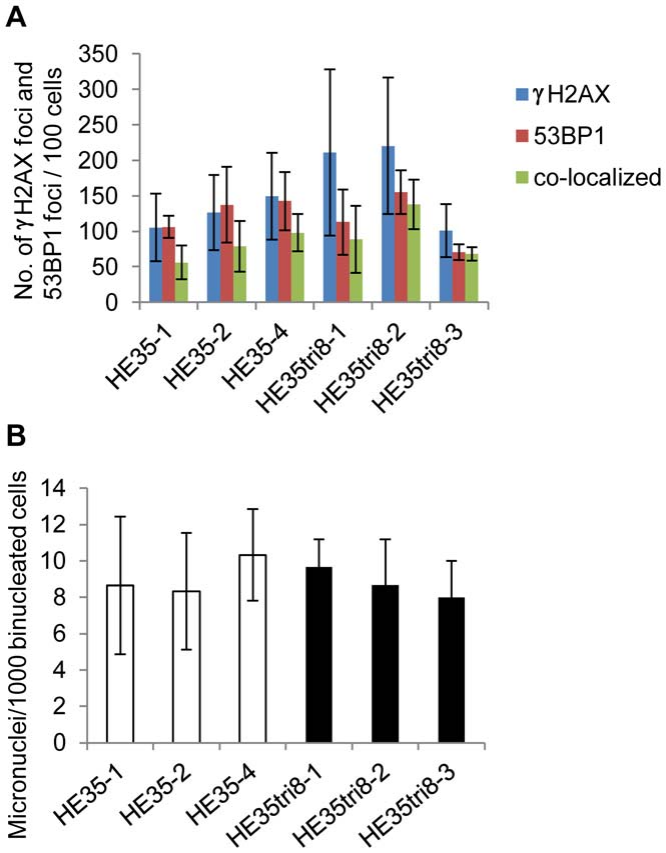
DNA damage and chromosome aberrations. (A) Number of γ-H2AX and 53BP1 *foci* in the diploid HE35 cells (HE35-1, -2, and -4) and the trisomy 8 cells (HE35tri8-1, -2, and -3). (B) Micronucleus frequency in the diploid HE35 cells and the trisomy 8 cells.

DNA double-strand breaks (DSBs) observed in early tumors initiate genomic instability that leads to cancer [16]. We examined whether DNA DSBs occurred in the trisomy 8 cells (HE35tri8-1, -2, and -3). Previously, both γ-H2AX and 53BP1 were shown to produce foci that co-localized with DSBs [17]–[20]. There were no differences in the number of foci containing co-localized γ-H2AX and 53BP1 in the trisomy 8 cells and the diploid HE35 cells (Figs. 3A, S4, S5).

Micronucleus assays have emerged as the preferred method to assess chromosome damage because micronuclei provide an index of both chromosome breakage and non-disjunction. Micronuclei are the origin of lagging whole chromosomes and acentric chromosome fragments during anaphase [21]. The micronucleus frequencies were no difference between the trisomy 8 cells and the diploid HE35 cells (Fig. 3B).

Chromosome aberrations are distinctive features of tumors [22]. To investigate whether trisomic conditions bring about chromosome aberrations, we analyzed metaphase chromosome aberrations. Diplochromosomes (chromosomes that have undergone DNA replication but have not segregated) were detected only in the trisomy 8 cells (Table 2, Fig. S6). Chromatid-type aberrations were observed in both the diploid HE35 cells and the trisomy 8 cells, whereas chromosome-type aberrations were found only in the trisomy 8 cells (Table 2). Although the trisomy 8 cells tended to induce both chromosome aberrations and DNA DSBs more than the diploid HE35 cells, there was not the statistical significance.

**Table 2:** Frequency of chromosome aberrations in the trisomy 8 cells.

Cells	Ploidy	No. of cells scored	Chromosome-type aberration (%)			Chromatid-type aberration (%)			Diplo-chromosome (%)
			Gap	Break	Ring	Gap	Break	SU*	
HE35-1	Diploid	50	0	0	0	0	0	0	0
HE35-2	Diploid	50	0	0	0	0	2	0	0
HE35-4	Diploid	50	0	0	0	0	2	0	0
HE35tri8-1	Trisomy 8	50	2	4	2	0	0	0	6
HE35tri8-2	Trisomy 8	50	0	2	4	4	0	0	8
HE35tri8-3	Trisomy 8	50	2	0	0	0	0	2	0

*Sister Union.

### Disruption of global gene expression patterns in the trisomy 8 cells

**Figure 4:**
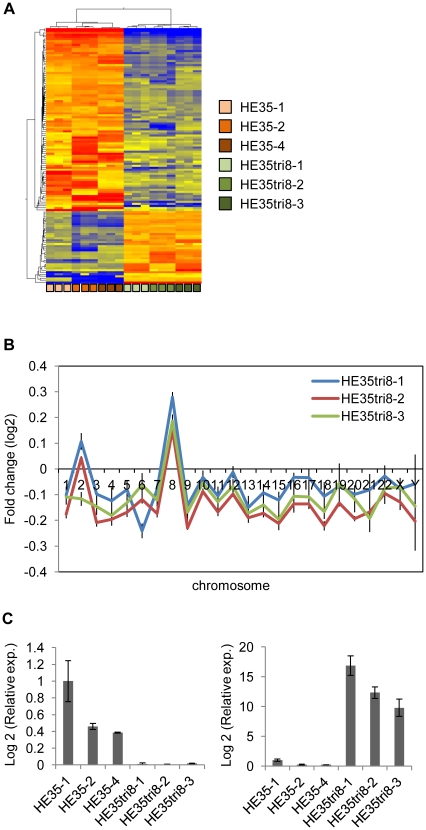
Disruption of the global gene expression patterns in the trisomy 8 cells. (A) Clustering analysis of 127 genes that exhibited statistically significant changes in the trisomy 8 cells (HE35tri8-1, -2, and -3) versus the diploid HE35 cells (HE35-1, -2, and -4). Three independent experiments were performed for each clone. (B) Average gene expression pattern by chromosome. The average chromosome expression ratios relative to the same reference RNA pool for each trisomy 8 cells was then normalized to the average of the ratio value for the diploid HE35 cells. (C) CADM1 (11q23.2) and WT1 (11p13) gene expression in the trisomy 8 cells (HE35tri8-1, -2, and -3) and the diploid HE35 cells (HE35-1, -2, and -4).

We compared the gene expression profiles of the trisomy 8 cells by microarray analysis. A clustering analysis of genes that exhibited statistically significant changes revealed a pattern that clearly separated the trisomy 8 cells from the diploid HE35 cells (Fig. 4A). The complete dataset is available at the NCBI GEO database (http://www.ncbi.nlm.nih.gov/projects/geo, accession number GSE28076).

The number of genes expressed on each chromosome was examined using a genome-wide transcript expression analysis. Total 127 genes were significantly altered in expression in the trisomy 8 cells compare with the diploid HE35 cells (Table S1). 72% of genes (91 genes) altered in expression were up regulated and the rest (36 genes) were down regulated. We did not yet identify a pathway associated with these genes. The pattern of genes expressed on each chromosome was similar in three clones of the trisomy 8 cells (HE35tri8-1, -2, and -3). The level of genes expressed on trisomic chromosome 8 was increased an average of 115% in each of the trisomy 8 cells compared to those of the diploid HE35 cells (Fig. 4B). In contrast, the average gene expression level on the other chromosomes was significantly decreased (Fig. 4B).

The trisomy 8 cells showed the malignant morphology, which occurs for a decrease of the cell adhesion ability. Therefore, to identify significantly deregulated cell adhesion genes that are involved in the loss of contact inhibition, microarray analyses were performed. As shown in Fig. 4C, Cell adhesion molecule 1 (CADM1) was dramatically down regulated in the trisomy 8 cells. Wilms tumor 1 (WT1), a known oncogene, was markedly up regulated in the trisomy 8 cells (Fig. 4C), but the relationship between WT1 and the phenomena in the trisomy 8 cells remained unclear. Although we have also done the pathway analysis, unfortunately there was no pathway related with 127 genes that changed more than 10 times.

## Discussion

Our analysis of normal human cells containing an additional chromosome 8 (trisomy 8 cells) revealed that all trisomy 8 cells share similar characteristics such as altered gene expression, proliferation defects, loss of contact inhibition, and regrowth after senescence. A small portion of the trisomy 8 cells regrew and formed colonies after 4–6 weeks of exhibiting a senescence-like state (Figs. 2C–D, Table 1). The trisomy 8 is the most commonly observed chromosome number aberration in fibroblastic/myofibro- blastic tumors (Fig. S1) [9]–[12]. Transformed cells lose contact inhibition [15] and do not undergo senescence. Our data showed that introducing a chromosome 8 into the normal diploid cells causes expression of transformation-associated phenotypes, such as chromosome instability, and malignant morphological characters. However, all trisomy 8 cells did not finally immortalize. We previously reported that human embryonic cells rapidly deplete telomerase activity associated with the significant shortening of telomeres, and then reached senescence. However, rodent embryo cells retained telomerase activity and the long telomeres (19–50 kb) during the long-term cultures, and cells immortalized. [23], [24]. It is likely that trisomy of chromosome 8 might not succeed reactivation of telomerase of human cells. However, unfortunately, because cell number collected from the regrowth colony was too few in the present study, we could not measure telomerase activity yet.

The number of foci where γ-H2AX and 53BP1 co-localized, and micronucleus were no difference between the trisomy 8 cells and the diploid HE35 cells (Fig. 3A–B). However, structural chromosome aberrations were increased in the trisomy 8 cells (Table 2). The number of foci where γ-H2AX and 53BP1 co-localized and the micronucleus frequencies were indirect method of DSB observation, whereas structural chromosome aberrations were direct method. This suggests that the trisomy does not cause the genetic instability due to DNA DSBs. And also these data suggested that DNA DSB is not a cause of becoming trisomy, but is a result of trisomy.

Diplochromosomes were found only in the trisomy 8 cells, suggesting that trisomy of chromosome 8 causes production of tetraploidy. Although chromatid-type aberrations were seen in both the diploid HE35 cells and the trisomy 8 cells, chromosome-type aberrations were observed in the trisomy 8 cells and not in the diploid HE35 cells. Increased chromosome-type aberrations, but not chromatid-type aberrations, have been associated with an increased cancer risk [25]. The present results revealed that trisomy of chromosome 8 causes other numerical and structural chromosomal aberrations that contribute to the relationship between increased chromosome instability and subsequent cancer risk.

Previous reports have shown that the average level of gene expression on trisomic chromosomes is increased in mouse cells, human cancer cells, and immortalized human cells compared to diploid cells [4]–[6]. Our artificial trisomy cells derived from primary human cells had greater average gene expression on the additional chromosome 8 (Fig. 4B). Surprisingly, the average gene expression level on all non-trisomic chromosomes was all decreased; moreover, the profile of each clone was similar (Fig. 4B). Total 127 genes were significantly altered in expression in the trisomy 8 cells compare with the diploid HE35 cells (Table S1). However, it is not clear whether this phenomenon is event that is specific for chromosome 8. The results of our pilot study show that similar change of gene expression is obtained in trisomy of chromosome 1, 6 and 7 (data not shown). These results strongly suggest that gaining even a single chromosome disrupts the expression levels on the trisomic chromosome as well as on the other chromosomes.

Each chromosome occupies a non-random and confined space in the interphase nucleus of higher eukaryotes [26]–[28]. There is increasing evidence that the positioning of genomic regions in the nuclear space is important for gene regulation [29]. One possible explanation for the general disturbances in the gene expression levels in the trisomy cells is alterations in chromosomal territory.

CADM1 was markedly down regulated in the trisomy 8 cells. The tumor suppressor CADM1 is involved in cell adhesion and is preferentially inactivated in invasive cancers [30]–[32]. CADM1 is expressed universally in human tissues and is frequently silenced in a variety of human carcinomas [33], [34]. A recent study further showed that hypermethylation of the CADM1 promoter induced gene silencing [33], [35]–[40]. We speculate that a down-regulation of the CADM1 gene in the trisomy 8 cells results from hypermethylation of the CADM1 promoter region.

In contrast, WT1 was significantly up regulated in the trisomy 8 cells. The WT1 gene was isolated as the gene responsible for a childhood renal neoplasm, Wilms' tumor, which is thought to arise due to the inactivation of both alleles of the WT1 gene located at chromosome 11p13 [41]–[43]. The WT1 gene was originally defined as a tumor suppressor gene [41], [44]–[48], but recent studies suggest that the WT1 gene is highly expressed in leukemia and solid tumors and likely plays an oncogenic role in leukemogenesis and tumorigenesis [49], [50]. It is possible that aneuploidy results in an epigenetic modification of WT1.

In conclusion, our findings strongly suggest that the addition of a single chromosome causes chromosome instability and extensive disruption of gene expression. Such critical and extensive changes in gene expression can produce parts of transformation-associated phenotypes in aneuploid cells.

## Materials and Methods

### Cells and cell culture

Human embryonic (HE35) cells were obtained from 7- to 8-week-old human embryos previously described [51]. HE35 cells were cultured in Eagle's Minimum Essential Medium (MEM) supplemented with 10% fetal bovine serum at 37°C with 5% CO_2_ in a humidified environment. The trisomy 8 cells (HE35tri8-1, -2, and -3 cells) were cultured in Eagle's Minimum Essential Medium (MEM) containing 800 µg/ml G418 supplemented with 10% fetal bovine serum at 37°C with 5% CO_2_ in a humidified environment. Briefly, both the diploid cells derived from HE35 (HE35-1, 2, and 4) and the artificial trisomy 8 cells (HE35tri8-1, -2, and -3) at TPDL 30 were plated at 5×10^5^ cells per T75 flask. Subconfluent cells were trypsinized and counted to determine the number of cells per T75 flask, and the cells were then replated at 5×10^5^ cells per T75 flask. Medium change has done every three days of all cultures. This process was repeated until there were either insufficient cells for plating or immortalization (as determined by increased cell proliferation).

### Microcell-mediated chromosome transfer

Donor mouse A9 cells containing human chromosome 8 were established by Kugoh et al. [52]. A9 cells grown in three T-25 flasks at 7×10^5^ cells/flask in medium containing 800 µg/ml G418 (Nakarai Tesque, Kyoto, Japan). To generate normal human cells containing an additional chromosome 8, we used microcell-mediated chromosome transfer (MMCT) procedure [13]. Briefly, the cells were incubated with 0.05 µg/ml colcemid in medium plus 20% fetal bovine serum for 48 h (to induce micronucleus formation), and then centrifuged in the presence of 10 µg/ml cytochalasin B (Sigma, MO, USA) at 8,000 rpm for 1 h at 34°C to isolate the micronuclei. The micronuclei were then purified by sequentially filtering through sterile filters of pore size 8-, 5-, and 3-µm. The purified micronuclei were suspended in fusion buffer and then HVJ-E was added (Genomone-CF; Ishihara Sangyo, Osaka, Japan) to the recipient cells (HE35 at TPDL7), which were kept on ice for 5 min, and then incubated at 37°C for 15 min. The supernatant was aspirated and MEM containing 800 µg/ml of G418 and 10% FBS was added.

We isolated three independent trisomy 8 cells (HE35tri8-1, -2, and -3) in 35-mm diameter dish (P35), and cells were cultured with doing stepwise scale up using P35, 60-mm diameter dish (P60), and 100-mm diameter dish (P100). Then, cells were cultured until reaching confluence in three P100 dishes. At this point, because total cell number in three dishes is approximately 8×10^6^, cells of each clone had divided at least 23 times (8×10^6^≈2^23^) during cloning process. Then, cells were stocked in liquid nitrogen until doing assay. Therefore, cells at 30 TPDL (7PDL+23PDL) were used for subsequent assay.

### Karyotype analysis

To prepare the metaphase chromosome, 5×10^5^ cells were seeded in P100 dishes. After incubating for 48 h, colcemid (Gibco, CA, USA) was added at a final concentration of 0.06 µg/ml, and the cells were treated for 2 h. Mitotic cells were collected and treated with 0.075 M potassium chloride for 25 min at room temperature. The cells were fixed in Carnoy's solution (methanol: acetic acid, 3∶1) and spread on glass slides using the air-drying method. After the cells were stained with a 3% Giemsa solution, the number of chromosomes with at least 50 metaphases per sample was scored.

### Multicolor fluorescence *in situ* hybridization (M-FISH)

Multicolor FISH was performed according to the manufacturer's protocol (Cambio, Cambridge, UK). A chromosome slide was aged on a hot plate at 65°C for 90 min, and the samples were denatured in a solution (70% formamide in 2X SSC) at 65°C for 2 min. After the reaction was quenched in ice-cold 70% ethanol for 4 min, the slides were dehydrated by washing for 5 min each in 70% ethanol and 100% ethanol and then dried at 37°C. An aliquot (10 ml) of the M-FISH probes was denatured at 65°C for 10 min and applied to the chromosome slide. Hybridization was performed at 37°C for 48 h in a humidified atmosphere. After hybridization, each slide was washed twice for 5 min each in washing solution (50% formamide in 0.5X SSC) at 45°C, followed by two incubations of 5 min each in 1X SSC at 45°C. Each slide was then incubated for 4 min in detergent washing solution (0.05% detergent DT in 4X SSC) at 45°C. An aliquot (125 ml) of the detection reagent was applied to the slides, which were then covered with parafilm and subsequently incubated in a humidified atmosphere for 20 min at 37°C. After the parafilm was removed, the slides were washed three times for 4 min in detergent washing solution at room temperature. Finally, the DNA was stained with 4′, 6-diamino-2-phenylindole (DAPI) in antifade solution. Chromosome images were captured and analyzed using the Leica CW4000 system.

### Proliferation assay

Exponentially growing diploid HE35 cells (HE35-1, -2, and -4) at TPDL 37 and the trisomy 8 cells (HE35tri8-1, -2, and -3) at TPDL 37 were plated at a density of 5×10^4^ cells in individual wells of multiple 6-well plates. All cells were plated in a final volume of 3 ml of medium. Cells were incubated in humidified 5% CO_2_ incubator at 37°C. The medium was replaced with fresh medium every three days throughout the experiment. Cells were harvested by trypsinization and cell number was counted by hemocytometer every day.

### Senescence-associated β-galactosidase assay

Cells were washed once with Ca^2+^- and Mg^2+^-free phosphate-buffered saline (PBS^−^) and fixed in a 0.2% paraformaldehyde solution containing 0.2% glutaraldehyde for 5 min at room temperature. After washing with PBS^−^, the fixed cells were incubated in SA-β-gal staining solution (40 mM citric acid/sodium phosphate, pH 6.0, 5 mM potassium ferrocyanide, 5 mM potassium ferricyanide, 150 mM NaCl, and 2 mM MgCl_2_) containing 1 mg/ml 5-bromo-4-chloro-3-indolylβ-D-galactopyranoside (X-gal) to stain senescent cells.

### Immunofluorescence detection

Both the diploid HE 35 cell and the trisomy 8 cells at TPDL 37 were fixed in 4% formaldehyde in PBS^−^ for 15 min, permeabilized for 10 min on ice in 0.5% Triton X-100 in PBS^−^, and then washed extensively with PBS^−^. Then, the coverslips were incubated with anti-phosphorylated histone H2AX at serine 139 (Upstate Biotechnology, NY, USA) and 53BP1 (Bethyl Laboratory, TX, USA) in TBS-DT (20 mM Tris-HCl, 137 mM NaCl, pH 7.6, containing 50 mg/ml skim milk and 0.1% Tween-20) for 2 h at 37°C. The primary antibodies were washed with PBS^−^, and Alexa Fluor 488–labeled anti-mouse IgG antibody and Alexa Fluor 594–labeled anti-rabbit *IgG* antibody (Molecular Probes, CA, USA) was added. The coverslips were incubated for 1 h at 37°C, washed with PBS^−^, and sealed on glass slides with 0.05 ml of PBS^−^ containing 10% glycerol. The cells were examined by fluorescence microscopy.

### Micronucleus assay

Cells at TPDL 37 were treated with 2 µg/ml cytochalasin B for 24 h in a T25 flask. They were then harvested and treated with 3 ml of hypotonic (0.1 M) KCl for 20 min, and fixed with 3 ml of methanol/acetic acid (5∶1). The cell suspensions were centrifuged at 1,200 rpm for 5 min. Then, the cells were suspended in 4 ml methanol/acetic acid solution and incubated on ice for 5 min. After centrifugation, the supernatant was removed and a 0.5–1 ml methanol/acetic acid solution was added to the cells. The cell suspensions were dropped onto slides and stained with 7.5% Giemsa for 40 min. The number of micronuclei per 1,000 binucleated cells was counted.

### Transcript array and date analysis

RNA was isolated from cells at TPDL 37 using an RNeasy Mini Kit (Qiagen, Tokyo, Japan). Five hundred nanograms of total RNA was then reverse-transcribed and labeled with a Quick Amp Labeling Kit, as recommended by the manufacturer (Agilent Technologies, CA, USA) and hybridized to Human Whole Genome Arrays (Agilent Technologies, CA, USA). Chips were analyzed and the data were extracted for examination using GeneSpring GX 11.5 Software (Agilent Technologies, CA, USA). To identify significantly related genes, GeneSpring GX 11.5 was used to perform a *t*-test.

### Accession number

The microarray data reported herein are available at the NCBI GEO database (http://www.ncbi.nlm.nih.gov/projects/geo, accession number GSE28076).

## Supporting Information

Figure S1 The distribution of trisomy in fibroblastic/myofibroblastic tumors (all sub types). The Mitelman Database of Chromosome Aberrations in Cancers was used as a source of the data (http://cgap.nci.nih.gov/Chromosomes/Mitelman).
(TIF)

Figure S2 The daily behavior of a culture of normal human cells (diploid HE35-1) and of a culture of the artificial trisomy 8 cells (HE35tri8-3) in the same region of culture. (A) normal human cells (diploid HE35-1), (B) artificial trisomy 8 cells (HE35tri8-3).
(TIF)

Figure S3 Senescence-associated β-galactosidase staining was altered in cultures of the diploid HE35 cells (HE35-1, -2, and -4). β-galactosidase staining is shown in blue.
(TIF)

Figure S4 The diploid HE35 cells (HE35-1, -2, and -4) were coimmunostained with anti-γ-H2AX and anti-53BP1 antibodies.
(TIF)

Figure S5 The trisomy 8 cells (HE35tri8-1, -2, and -3) were coimmunostained with anti-γ-H2AX and anti-53BP1 antibodies.
(TIF)

Figure S6 Diplochromosomes at metaphase in the trisomy 8 cell (HE35tri8-1).
(TIF)

Table S1 The gene number that an expression level was significantly changed by a chromosome 8 introduction.
(TIFF)
