# Supplementary figures and images for: Dysregulation of Gene Expression in the Artificial Human Trisomy Cells of Chromosome 8 Associated with Transformed Cell Phenotypes
Source: PLoS One. 2011 Sep 29;6(9):e25319. doi: 10.1371/journal.pone.0025319 (PMC3183047; doi:10.1371/journal.pone.0025319)

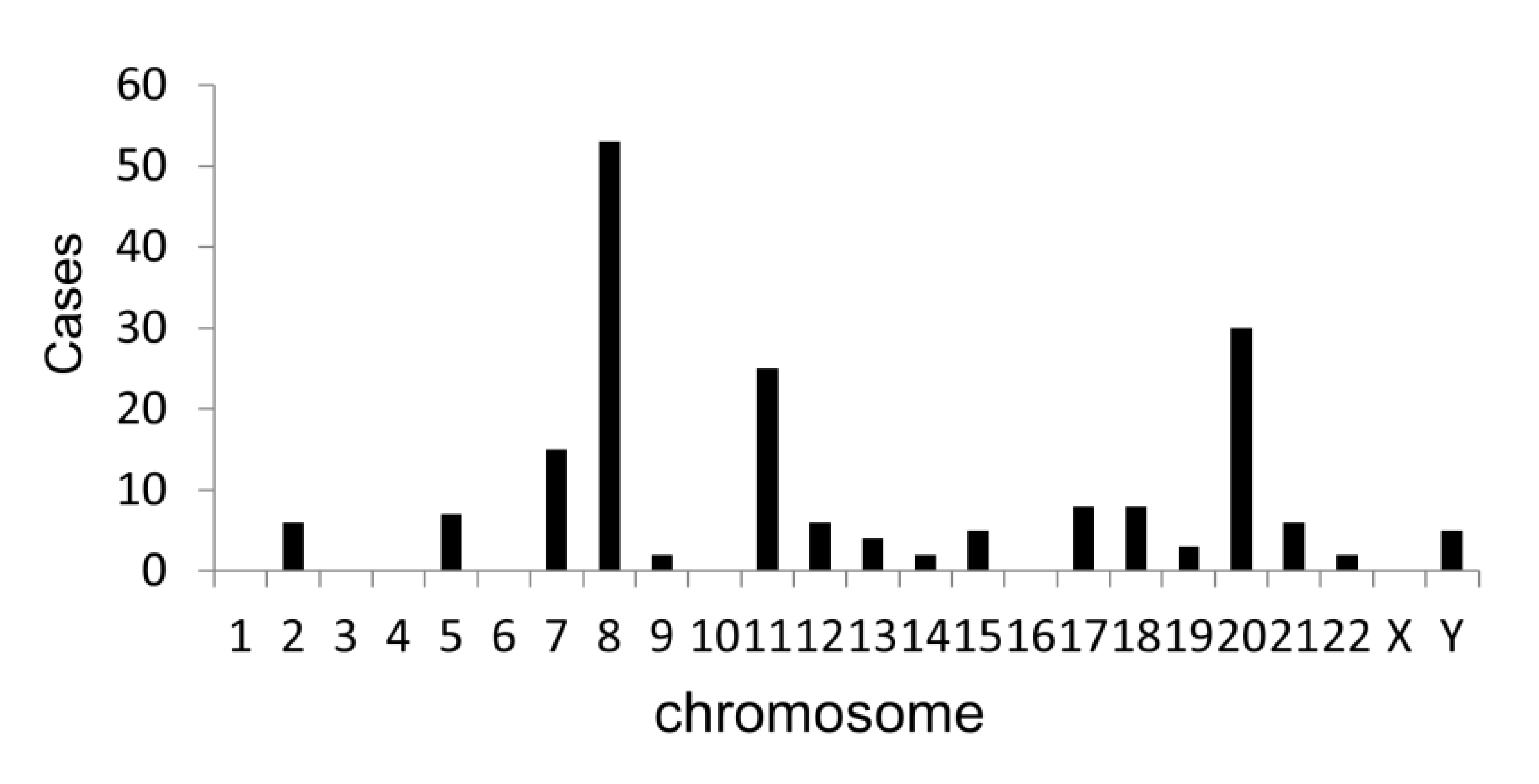

Supplement: Figure S1 — The distribution of trisomy in fibroblastic/myofibroblastic tumors (all sub types). The Mitelman Database of Chromosome Aberrations in Cancers was used as a source of the data (http://cgap.nci.nih.gov/Chromosomes/Mitelman). (TIF) [file pone.0025319.s001.tif]

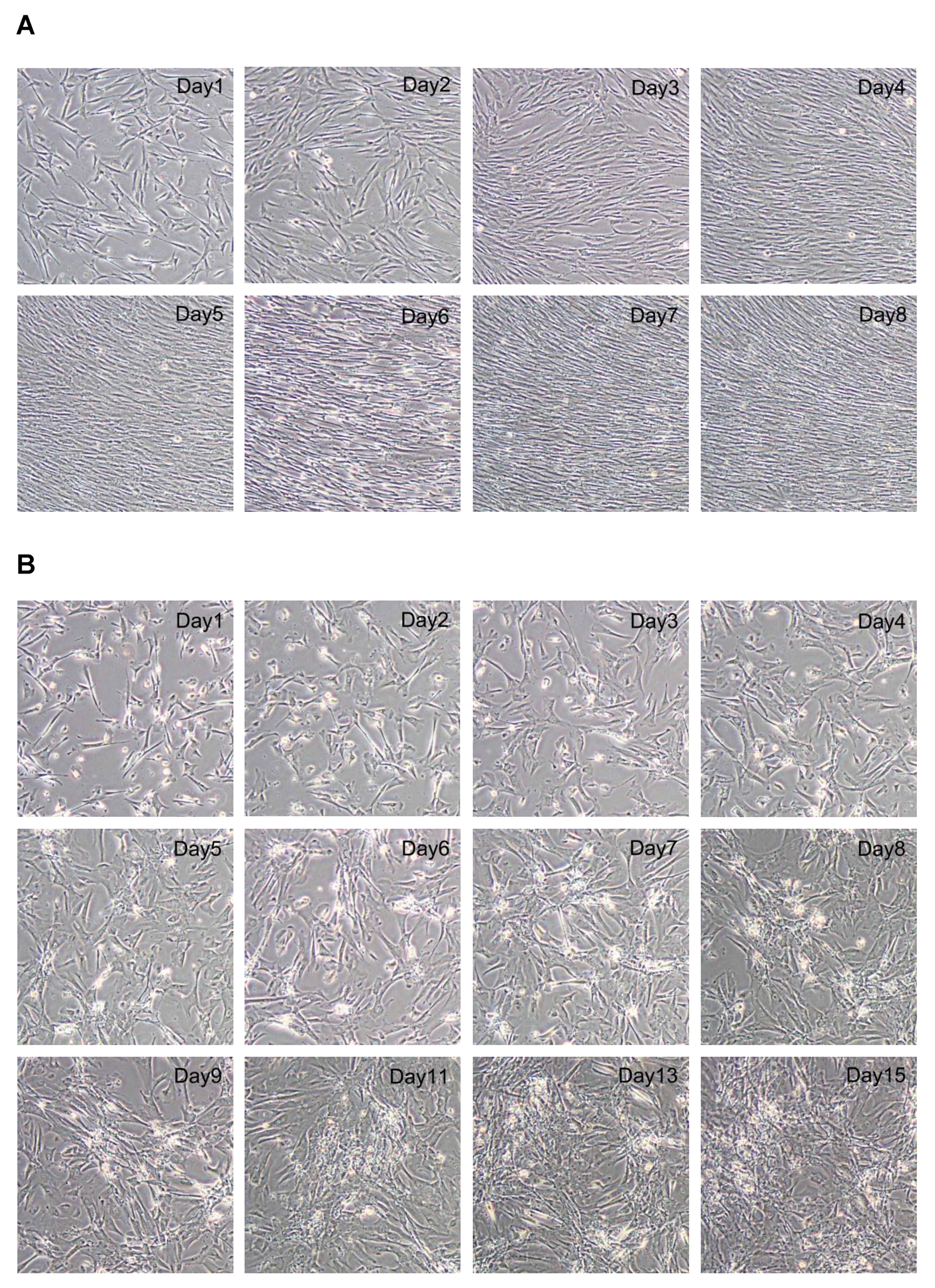

Supplement: Figure S2 — The daily behavior of a culture of normal human cells (diploid HE35-1) and of a culture of the artificial trisomy 8 cells (HE35tri8-3) in the same region of culture. (A) normal human cells (diploid HE35-1), (B) artificial trisomy 8 cells (HE35tri8-3). (TIF) [file pone.0025319.s002.tif]

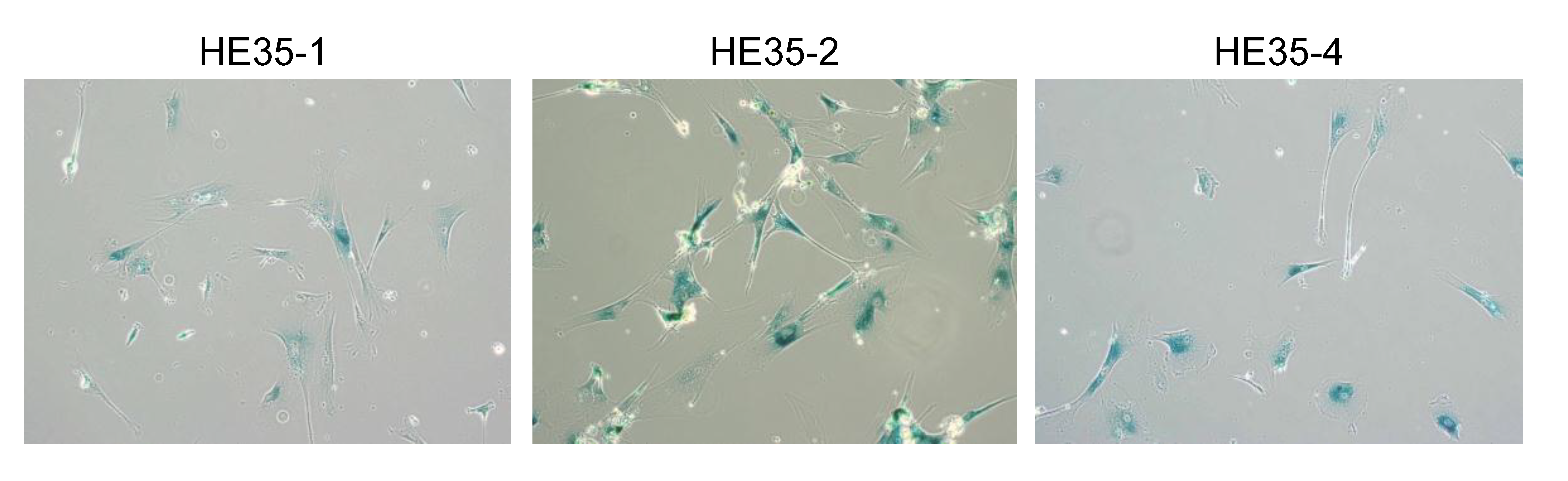

Supplement: Figure S3 — Senescence-associated β-galactosidase staining was altered in cultures of the diploid HE35 cells (HE35-1, -2, and -4). β-galactosidase staining is shown in blue. (TIF) [file pone.0025319.s003.tif]

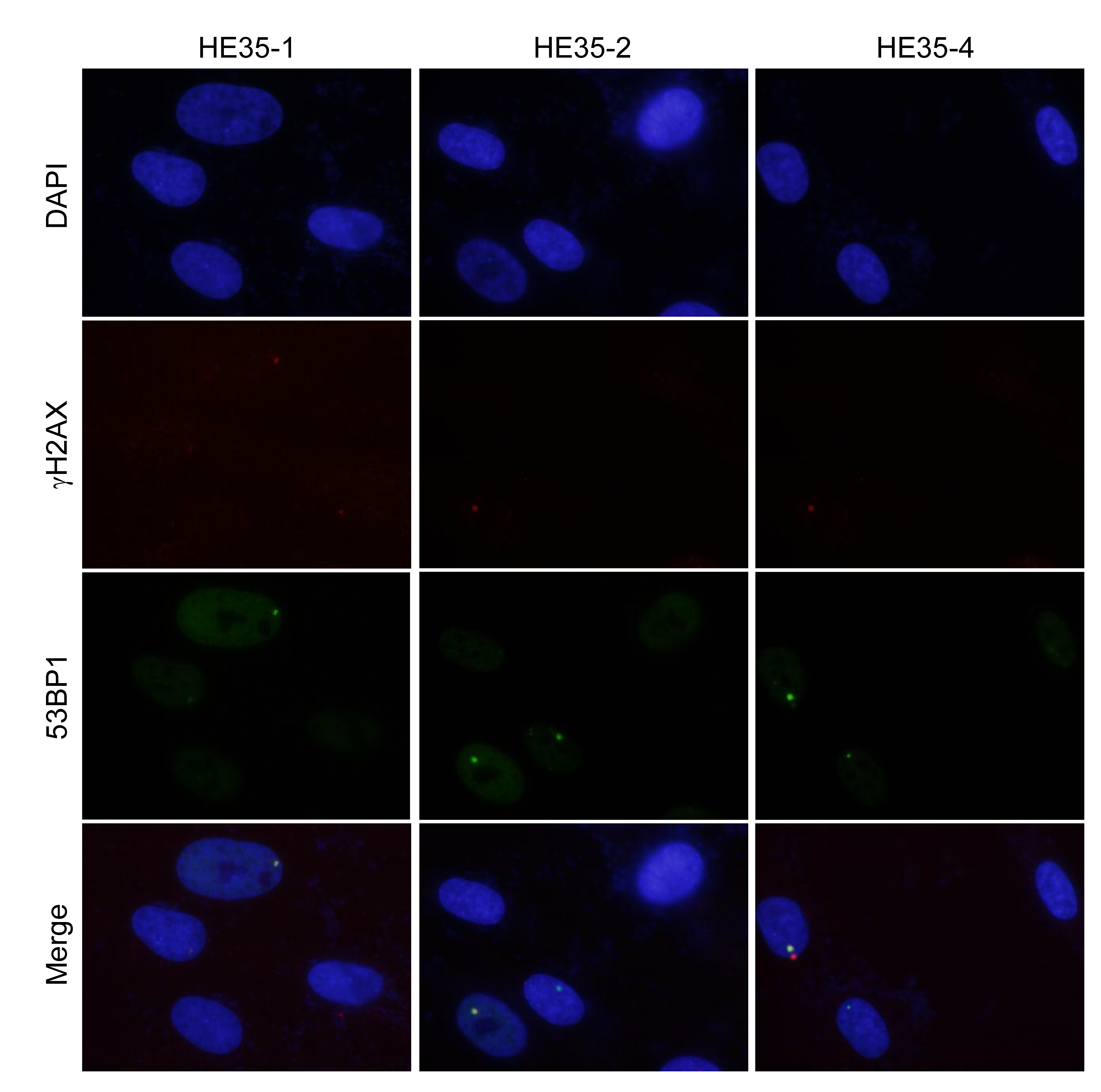

Supplement: Figure S4 — The diploid HE35 cells (HE35-1, -2, and -4) were coimmunostained with anti-γ-H2AX and anti-53BP1 antibodies. (TIF) [file pone.0025319.s004.tif]

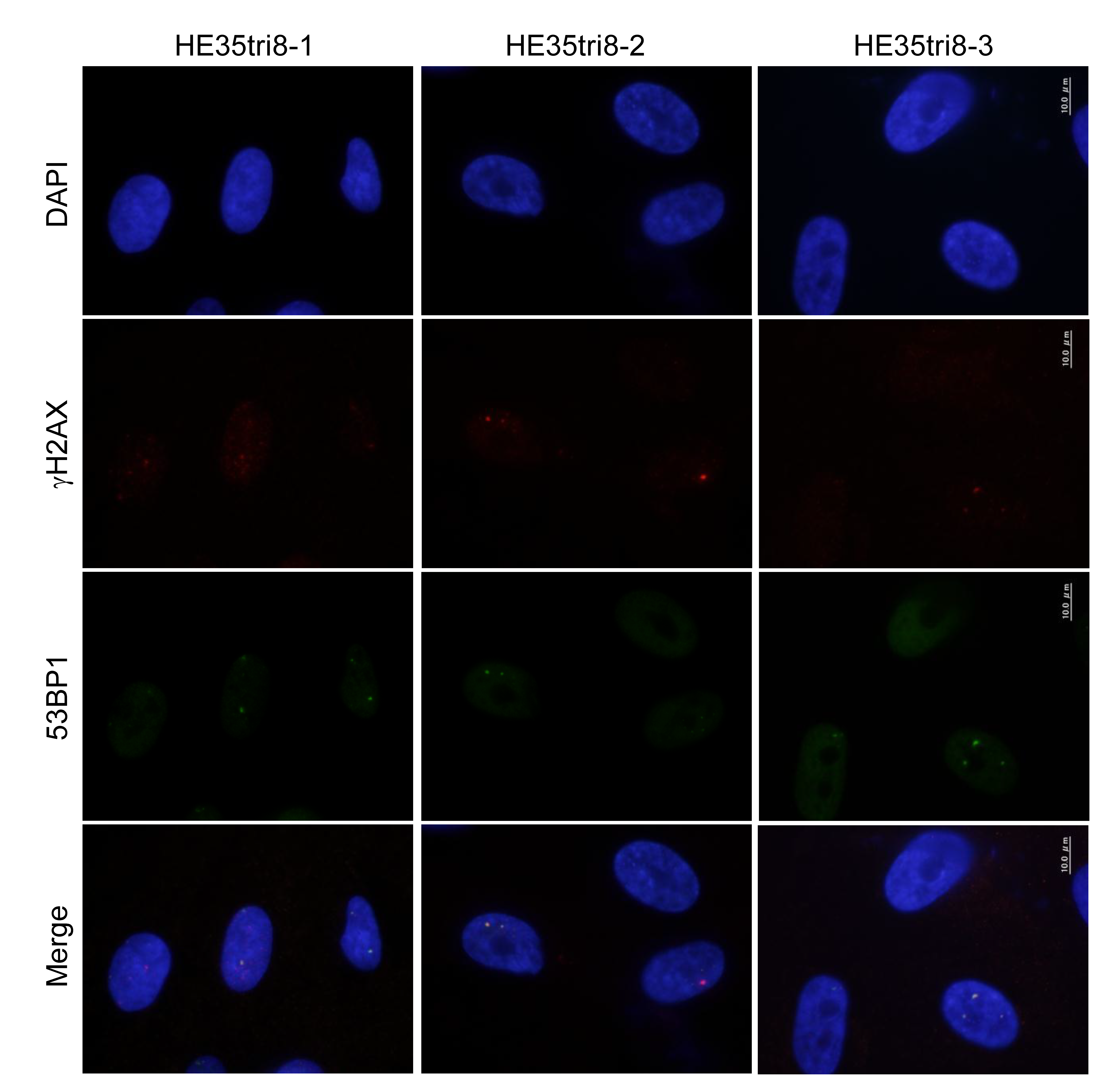

Supplement: Figure S5 — The trisomy 8 cells (HE35tri8-1, -2, and -3) were coimmunostained with anti-γ-H2AX and anti-53BP1 antibodies. (TIF) [file pone.0025319.s005.tif]

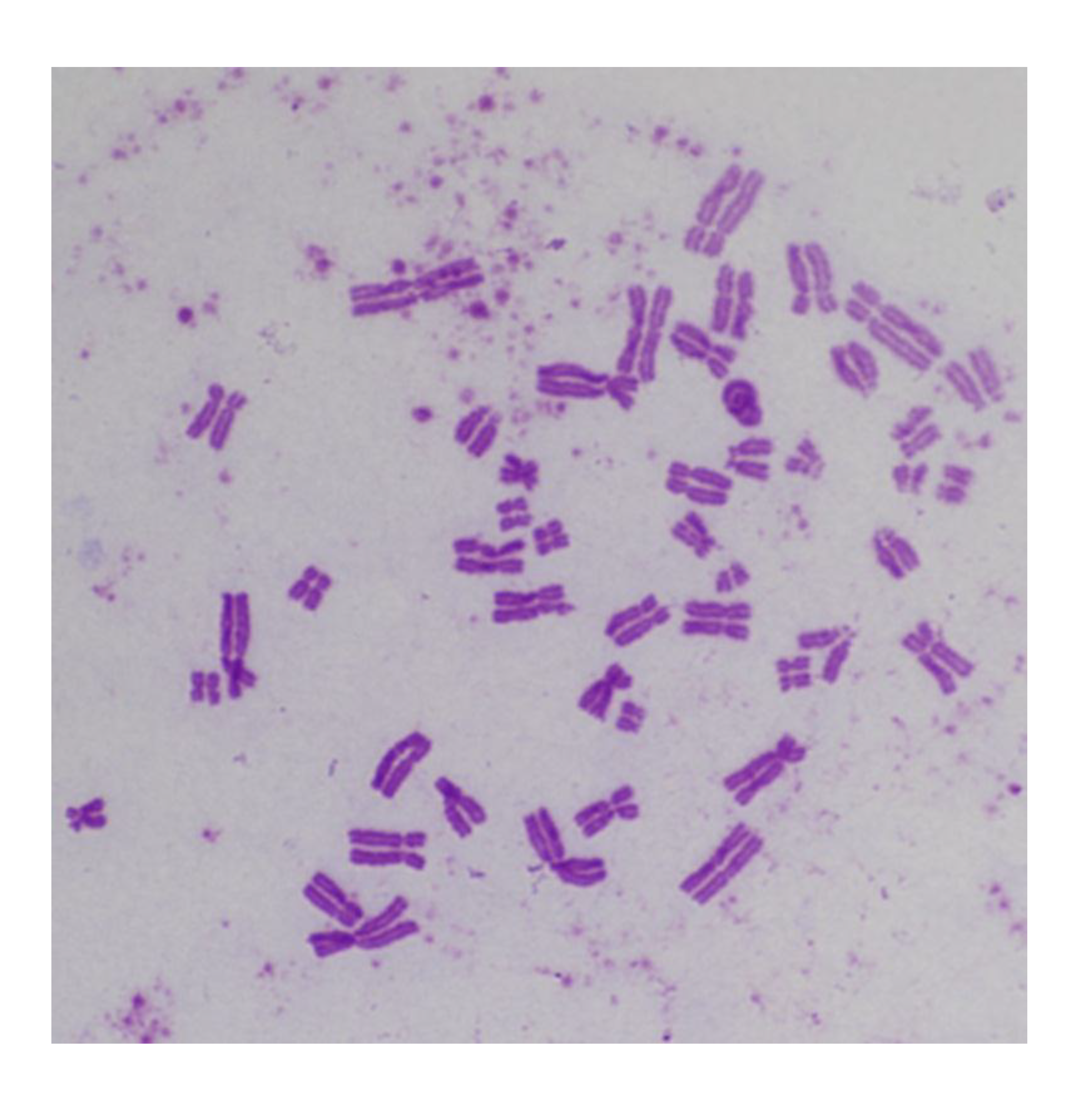

Supplement: Figure S6 — Diplochromosomes at metaphase in the trisomy 8 cell (HE35tri8-1). (TIF) [file pone.0025319.s006.tif]

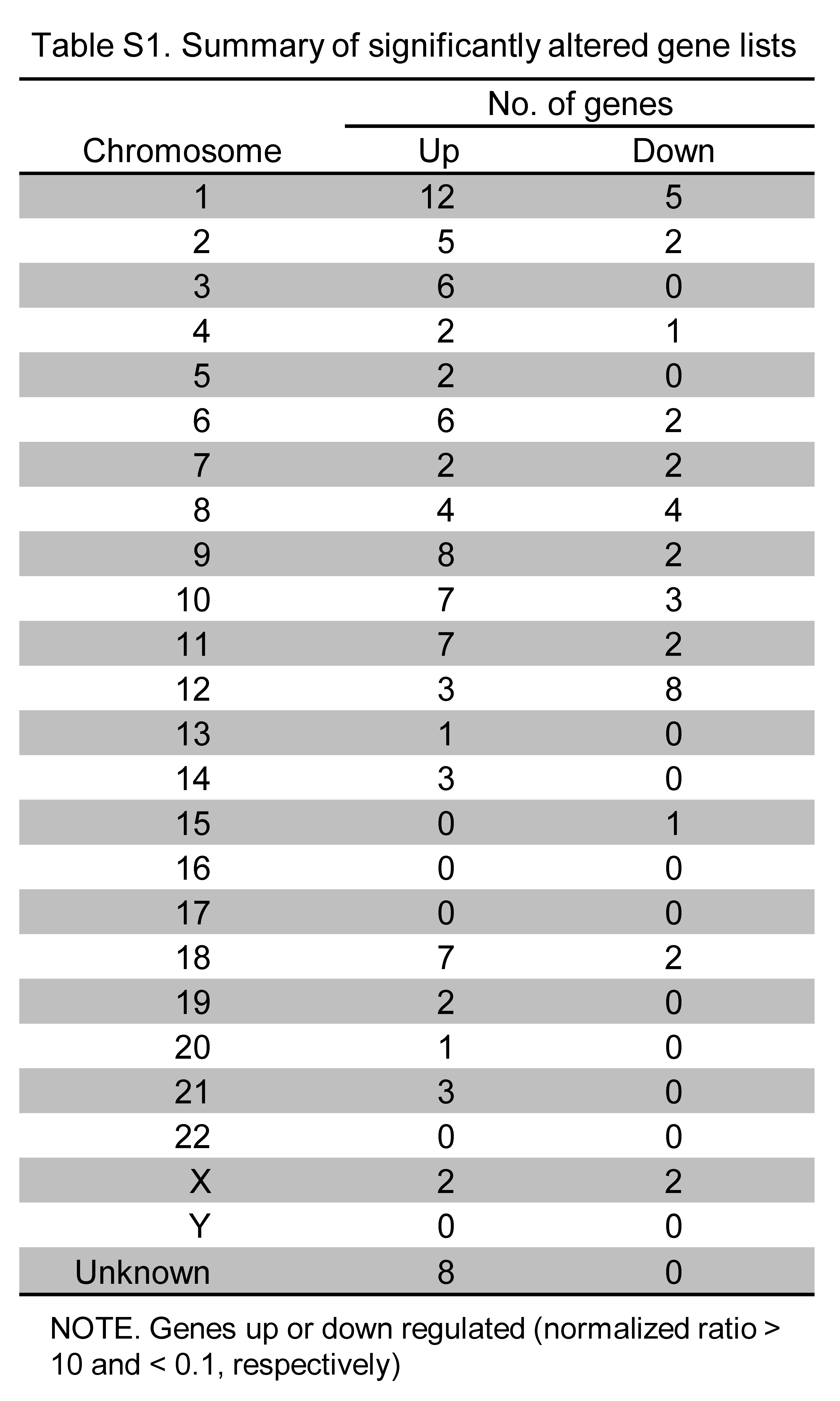

Supplement: Table S1 — The gene number that an expression level was significantly changed by a chromosome 8 introduction. (TIFF) [file pone.0025319.s007.tif]
